# An in vitro model for hypertrophic adipocytes: Time‐dependent adipocyte proteome and secretome changes under high glucose and high insulin conditions

**DOI:** 10.1111/jcmm.15497

**Published:** 2020-07-03

**Authors:** Qi Qiao, Freek G. Bouwman, Johan Renes, Edwin C. M. Mariman

**Affiliations:** ^1^ Department of Human Biology NUTRIM School of Nutrition and Translational Research in Metabolism Maastricht University Medical Centre Maastricht The Netherlands

**Keywords:** cell metabolism, extracellular matrix, overfeeding, proteome, secretome, SGBS adipocytes

## Abstract

Obesity is the consequence of a positive energy balance and characterized by enlargement of the adipose tissue, which in part is due to hyperplasia and hypertrophy of the adipocytes. Not much is known about the transition of normal mature adipocytes to the hypertrophic state, which in vivo is very hard to study. Here, we have maintained mature human SGBS cells as a surrogate for adipocytes, changes of morphological and molecular metabolism of the adipocytes were monitored over the first 4 days and the last 4 days. In total, 393 cellular proteins and 246 secreted proteins were identified for further analysis. During the first 4 days of high glucose and insulin, the adipocytes seemed to prefer pyruvate as energy source, whereas beta‐oxidation was down‐regulated supporting lipid loading. Over time, lipid droplet fusion instead of lipid uptake became relatively important for growth of lipid droplets during the last 4 days. Moreover, ECM production shifted towards ECM turnover by the up‐regulation of proteases over eight days. The present in vitro system provides insight into the metabolic changes of adipocytes under conditions of high glucose and insulin, which may help to understand the process of in vivo adipocyte hypertrophy during the development of obesity.

## 1 INTRODUCTION

Overweight/obesity is major risk factors for various health complications like type II diabetes, cardiovascular diseases and certain types of cancer^1^ and therefore provide a serious burden to health and social healthcare systems. To date, the prevalence of overweight/obesity is still increasing and not a single country has successfully reversed its epidemic.^2^ According to the World Health Organization, obesity is characterized by ‘abnormal or excessive fat accumulation’.^3^ Adipose tissue (AT) is the main lipid storage depot and plays a central role in buffering the daily balance between energy intake and energy expenditure.^4^ Excess energy intake by overeating is one of the causes of obesity.^5^ It leads to an increase in body weight,^6^, ^7^ body fat mass,^5^, ^7^, ^8^, ^9^ fat‐free mass,^5^ energy expenditure^10^ and AT remodelling by adipocyte hypertrophy or hyperplasia.^11^ Hypertrophy is accompanied by adipocyte dysfunction with disturbance of the lipid handling processes.^12^ In addition, obesity development is often paralleled by a decrease of whole body insulin sensitivity characterized by increased plasma levels of glucose and insulin, which is ascribed to the limits of AT expandability accompanied by ectopic lipid deposition.^13^, ^14^, ^15^

Human intervention studies by overfeeding (OF) are a way to shed light on the cellular and metabolic changes of the AT in relation to the development of obesity.^13^ Gene expression analysis indicated that after 7 or 28 days OF, changes in the expression profile of AT already can be observed.^14^, ^16^ Recently, Alligier et al showed that on OF subcutaneous AT shows different responses over time. Changes of genes expression after 14 days OF indicated a significant impact on the lipid metabolism and storage pathways, whereas after 56 days of OF changes related more to pathways of extracellular matrix (ECM) and inflammation.^15^

Another approach to study changes in cellular and metabolic behaviour of AT during OF is to use an in vitro culture system. Although such a model system does not reflect the in vivo situation directly, valuable clues to biological processes could be obtained, which specifically pertain to the development of hypertrophic adipocytes. Simpson Golabi Behmel Syndrome (SGBS) cells have been well accepted as an excellent in vitro surrogate for human white subcutaneous adipocytes with similarity in morphology, physiology and biochemistry.^17^, ^18^, ^19^, ^20^ Here, we subjected mature SGBS adipocytes to a high glucose and high insulin condition for 4 and 8 days and studied the changes of the cellular and secreted proteome. Comparing the changes of the first 4 days with those of the second 4 days OF showed a clear shift in the cellular processes.

## 2 MATERIALS AND METHODS

### 2.1 Cell culture

Human SGBS (pre)adipocyte culturing has been described in detail previously.^21^ In short, pre‐adipocytes of passage 9 were seeded in 6‐well plates (Corning, Sigma‐Aldrich, Zwijndrecht, the Netherlands) with 3 × 10^4^ cells per well. The culture medium was Gibco™ Dulbecco's Modified Eagle Medium: Nutrient Mixture F‐12 (DMEM/F‐12 (1:1); Life Technologies) supplemented with 66 mmol/L biotin, 34 mmol/L D‐pantothenate (Sigma‐Aldrich), 10% foetal calf serum (Bodinco BV, Alkmaar, the Netherlands) and 1% penicillin and streptomycin (Life Technologies). Once pre‐adipocytes reached a confluence of about 90%, it was shifted to differentiation procedure as previously described.^21^ In detail, the medium was changed to serum‐free DMEM/F12 differentiation medium containing 2 mg/mL human transferrin, 200 µmol/L human insulin, 5 mmol/L cortisol, 20 µmol/L triiodothyronine, 1 mmol/L 3‐isobutyl‐1‐methylxanthine and 5 mmol/L rosigilitazone (Sigma‐Aldrich). After 4 days, the medium was changed to serum‐free DMEM/F12 medium containing 2 mg/mL human transferrin, 200 µmol/L human insulin, 5 mmol/L cortisol and 20 µmol/L triiodothyronine. Every second day, the medium was refreshed. After 14 days, 85%‐88% of pre‐adipocytes were differentiated into mature adipocytes.

Since day 14, the medium of the mature adipocytes was changed to DMEM/F12 (1:1) without phenol red (Cell Culture Technologies), which contains 20 nmol/L human insulin and 17.5 mmol/L glucose. Cells were cultured for another 8 days, and samples for the cellular proteome and secretome were taken at T14 (time‐point of day 14), T18 (time‐point of day 18) and T22 (time‐point of day 22).

### 2.2 Morphology monitoring and Oil Red O staining

The morphology changes from day 14 onwards were closely recorded using a Nikon Eclipse TS100 microscope equipped with a Digital Sight microscope camera control unit (DS‐L3, Nikon). The mean diameter of the five biggest fat droplets was recorded and Oil Red O (ORO) staining was performed as parameters to monitor the turnover of the stored fat as previously described.^21^

### 2.3 Protein sample collection

For secreted protein isolation, the medium was collected at T14, T18 and T22 from each well separately. The collected medium (4 mL per well) was centrifuged at 2660 *g*  for 10 minutes (Universal 30 RF, Hettich Benelux BV, the Netherlands). Thereafter, the supernatant was gently transferred to a new tube, snap‐frozen in liquid nitrogen and stored at −80°C for further analysis.

For cellular protein collection, wells with cultured cells were washed twice with PBS buffer and lysed with SDT buffer (2% sodium dodecyl sulphate/50 mmol/L dithiothreitol/100 mmol/L Tris‐HCl pH = 7.6), 300 µL per well. Cells were scraped off with scraper (Corning) and the lysate was collected in tubes, then heated at 95°C for 5 minutes. After heating, samples were sonicated in three 20‐seconds cycles and centrifuged at 16 000 *g* for 5 minutes at 20°C, and then, the supernatant was carefully transferred to another tube. All samples were stored at −80°C for protein digestion and LC‐MS/MS quantification. The entire experiment was performed three times and for each experiment triplicates were available for each protein isolation.

### 2.4 Cellular sample preparation for LC‐MS/MS

Amicon Ultra 0.5‐mL centrifugal filter devices (Sigma‐Aldrich) were pre‐treated by soaking overnight in 5% Tween 20, washing with Milli‐Q for 10 minutes with 600 rpm shaking, repeat the washing by centrifuge at 14 000 *g* at 20°C for 25 minutes before use.

Cellular protein sample digestion has been described in detail previously.^21^ In short, after induction and alkylation, 42 µg protein of time point T14, T18 and T22 was supplemented with 1 µg trypsin/Lys‐C Mix (Thermo Fisher Scientific) and incubated for 9‐14 hours at 37°C. After overnight digestion, the peptide samples were cleaned from residual sodium deoxycholate and SDS by precipitation with an equivalent volume of 4 mol/L potassium chloride, acidified to pH = 1‐2 with 100% formic acid (FA). Then, peptide samples were desalted with a column made by stacking three layers of a 3 mol/L Empore C18 column (Thermo Fisher Scientific) in a P20 pipet tip. After the column was pre‐rinsed with 50 µL 70% acetonitrile (ACN) and equilibrated with 50 µL 100% FA by air pressure, the cellular samples were loaded on the column and eluted with 30 µL 70% ACN/5% FA, and the desalted sample was collected in a clean LoBind tube (Eppendorf, Sigma‐Aldrich). Peptides were dried under vacuum and labelled with TMT 10plex Mass Tagging Kits (Thermo Fisher Scientific; 90111) according to the manufacturer's protocol. In short, 42 µg cellular peptides diluted into 84 µL of 50 mmol/L triethyl ammonium bicarbonate were transferred into the reaction tube, which contained the labelling reagents dissolved in 41 µL anhydrous acetonitrile per tube. The labelling reaction was incubated for 1 hour at room temperature and quenched 15 minutes by adding 8 µL of 5% hydroxylamine. Equal amounts of combined samples were transferred into a new micro‐centrifuge tube for LC‐MS/MS with a final concentration of 0.33 µg/µL. The entire experiment was performed three times and as such generated nine samples per time point.

### 2.5 Secretome samples preparation for LC‐MS/MS

For secreted proteins, the digestion was on the filter.^22^ In general, the medium of each vial was added to the pre‐rinsed filter device, centrifuged at 4000 *g* at 20°C for 30 minutes. The concentrated sample on the filter was washed with 3.5 mL of 50 mmol/L ammonium bicarbonate and centrifuged at 4000 *g* at 20°C for 30 minutes. For reduction, 15 µL of 200 mmol/L dithiothreitol was added and the filter was incubated at room temperature for 45 minutes. Next, to accomplish alkylation 18 µL of 400 mmol/L iodoacetamide solution was added and incubated in darkness for another 45 minutes. To stop the alkylation, 30 µL of 200 mmol/L dithiothreitol was added and incubated for 45 minutes. Then, samples on the filter were washed once with 50 mmol/L ammonium bicarbonate at 4000 *g* at 20°C for 40 minutes. Subsequently, trypsin/Lys‐C was added in the ratio of 1 µg/25 µL sample. After gentle mixing, the filter device was incubated at 37°C overnight. Peptide concentration was measured by the Pierce Quantitative Colorimetric Peptide Assay according to the manufacture's protocol (Thermo Fisher Scientific, #23275). Then, digested peptides were diluted to the final concentration of 0.25 μg/μL with 50 mmol/L ammonium bicarbonate. The whole experiment was performed three times with triplicate samples. The triplicate samples from the first‐time experiment were pooled to serve proper protocol assessment. Therefore, totally seven samples were generated per time point from the three independent experiments. Compared with cellular proteins, the peptide concentration of the secretome samples was much lower, which made the secretome quantification more challenging and more sensitive method was needed. As TMT labelling yields lower peptide identification rates^23^ and lowered accuracy when quantified on MS^2^ level,^24^ therefore, TMT labelling was omitted and label‐free quantification was used for secretome samples.

### 2.6 Protein identification using LC‐MS/MS

A nanoflow HPLC instrument (Ultimate 3000, Dionex) was coupled online to a Q Exactive mass‐spectrometer (Thermo Fisher Scientific) with a nano‐electrospray Flex ion source (Proxeon). For cellular samples, 5 µL of the TMT labelled cellular peptide samples were loaded. For secreted samples, an equal amount of Pierce Digestion Indicator peptides was added to all peptide samples as internal standard, and then, 5 µL of this mixture were loaded as well. Samples were loaded onto a C18‐reversed phase column (Acclaim PepMap C18 column, 75‐μm inner diameter × 15 cm, 2‐μm particle size). The peptides were separated with a 120 minutes linear gradient of 4%‐68% buffer B (80% acetonitrile and 0.08% FA) at a flow rate of 300 nL/min.

MS data were acquired using a data‐dependent top‐10 method, dynamically choosing the most abundant precursor ions from the survey scan (280‐1400 *m/z*) in positive mode. Survey scans were acquired at a resolution of 70,000 and a maximum injection time of 120 mseconds Dynamic exclusion duration was 30 seconds. Isolation of precursors was performed with a 1.8 *m/z* window and a maximum injection time of 200 mseconds. Resolution for HCD spectra was set to 30,000, and the normalized collision energy was 32 eV. The under‐fill ratio was defined as 1.0%. The instrument was run with peptide recognition mode enabled, but exclusion of singly charged ions and charge states of more than five.

The MS data were searched using Proteome Discoverer 2.2 Sequest HT search engine (Thermo Fisher Scientific) against the UniProt human database. The false discovery rate was set to 0.01 for proteins and peptides, which had to have a minimum length of six amino acids. The precursor mass tolerance was set at 10 ppm, the fragment tolerance at 0.02 Da and one miss‐cleavage was allowed. For secreted and cellular samples, oxidation of methionine was set as a dynamic modification and carbamidomethylation of cysteines as fixed. TMT reagent adducts (+229.162932 Da) on lysine and peptide amino termini were set as fixed modifications for cellular samples.

### 2.7 Protein quantification

For cellular proteins, quantification followed relative comparison of the TMT‐specific peaks in the MS^2^ spectrum. Label‐free quantitation was conducted for secretome samples using the Minora Feature Detector node in the processing step and the Feature Mapper node combined with the Precursor Ions Quantifier node in the consensus step with default settings within Proteome Discoverer 2.2 (Thermo Fisher Scientific, XCALI‐97808).

### 2.8 Data normalization

For cellular proteins, the data of each run were normalized to the total peptide amount in each channel and to compare between the runs scaled to time point T18.

For secreted proteins, the LC‐MS analysis was done in seven runs, each run containing a sample from each time point (T14, T18 and T22). The total number of proteins that was identified in the medium was 1264. Data normalization was performed in two steps. First, to correct data for possible differences between runs, we chose the 476 proteins, which were present in all of the analysed samples. We calculated the mean abundance of those 476 proteins in all 7 runs (M) and also mean abundance per run (m_x_ for run x). Normalization factor 1 for run x (f1_x_) = M ÷ m_x_. Data were corrected (D1) as follows: D1 = f1_x_ × original protein abundance in run x. Also, the Pierce Indicator added to each sample was normalized by f1. The second normalization was then performed to stratify the protein abundances according to the Pierce Indicator. Normalization factor 2 for sample y (f2_y_) = Pierce's mean abundance from all samples ÷ Pierce abundance in sample y. In general, the second normalization step was: D2 = f2 × D1.

### 2.9 Validation of secreted proteins

To verify the secreted nature of the identified proteins, their amino acid sequences were obtained from UniProt and analysed with SignalP^25^, ^26^ and Deeploc.^27^ Proteins identified to contain a signal peptide by SignalP or validated in the extracellular space by Deeploc were picked up as secreted proteins. Accordingly, around 26.58% of the identified proteins from the medium was finally confirmed as secreted proteins.

### 2.10 Missing value handling

Performing LC‐MS analysis of proteins, values could be missing for various reasons.^28^ For cellular proteins, only those having no more than four missing values per time point were selected. As for secreted proteins, only proteins recognized as secreted and with no more than three missing values were selected. The Multiple Imputation routine of SPSS was used to impute those selected protein's missing values, and further analysis was subsequently performed.

### 2.11 Western blotting

The protein concentration was determined as described before by using the BCA kit (Pierce, Thermo Fisher Scientific; 23252). Fifteen microgram of extracted proteins was run on a 12% SDS‐PAGE gel, then electro‐transferred onto nitrocellulose membranes. After blocking with 4% non‐fat milk for 2 hours, the nitrocellulose membranes were incubated overnight at 4°C with primary antibodies against Akt and p‐Akt (AKT #9272, p‐AKT#9271, all 1:1000, Cell Signaling Technology). After washing three times with Tris‐buffered saline with 0.1% Tween 20 (TBST), each time for 10 minutes, the membranes were incubated with horseradish peroxidase (HRP) conjugated secondary antibodies (anti‐Rabbit DAKO cat# P0399) for 1 hour at room temperature. Then, after 3 × 10 minutes washing with TBST and 1 × 10 minutes with TBS alone, the protein blots were visualized with ECL detection reagent (Pierce; SuperSignal^TM^ west Dura femto max sensitivity; Thermo Fisher Scientific; 34095). The density of protein bands was determined and quantified with local background correction by using the ChemiDoc XRS system (Bio‐Rad).

### 2.12 Statistical analyses

Data were described as mean ± SEM (the standard error of the mean, SEM), and both cellular and secreted protein abundances were log_2_‐transformed. Proteome changes were analysed by using two‐tailed dependent *t* test with a cut‐off for significance of *P* < .05. Statistical analyses were conducted using SPSS version 22.0.

### 2.13 Functional analysis

Clustering of protein interactions were visualized by STRING.^29^ Enriched pathways were analysed by DAVID,^30^ and significantly changed cellular proteins and secreted proteins were pooled together to detect potentially affected processes by early feeding or late feeding.

## 3 RESULTS

### 3.1 Morphologic characteristics of SGBS cells during eight days feeding

**FIGURE 1:**
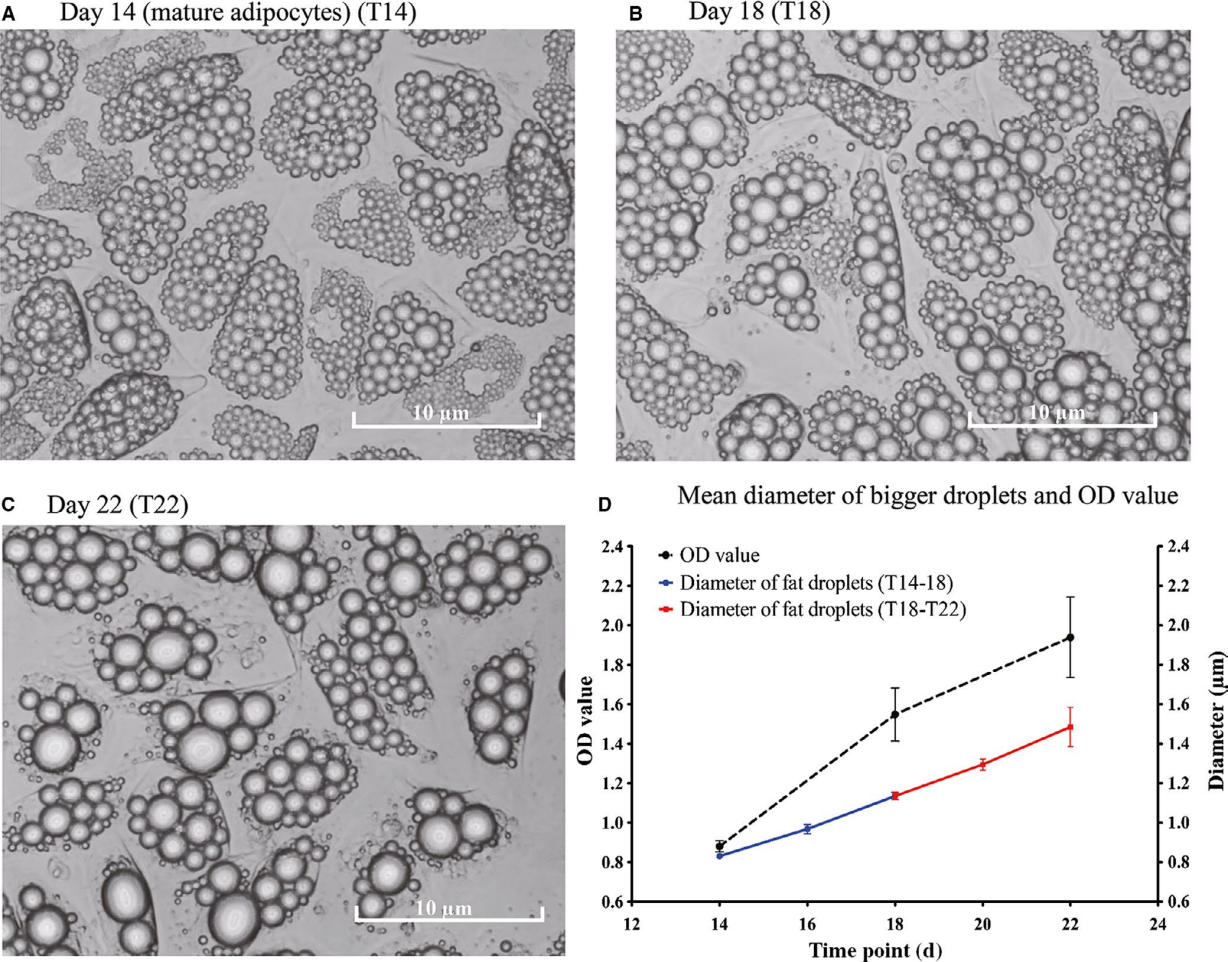
Recording of adipocytes morphological changes since day 14. (A) Morphology of day 14 mature SGBS adipocytes, (B) after initial four days feeding (T18), and (C) after eight days feeding (T22). (D) Lipid accumulation was measured by Oil Red O as well as the mean diameter of the 5 biggest lipid droplets during T14, T18 and T22

After 14 days differentiation, approximately 85%‐90% SGBS pre‐adipocytes had differentiated into mature adipocytes, which were largely occupied by fat droplets. The morphologic changes are recorded in Figure 1. During the early feeding period (T14‐T18), the adipocytes did not change in size, while the diameter of fat droplets increased. During the late feeding period (T18‐T22), the size of the bigger fat droplets continued to increase, while the number of visible fat droplets per cell decreased. Figure 1D shows the mean diameter of the five biggest fat droplets per cell over time.^21^, ^31^ The diameter of biggest fat droplets increased 0.30 µm after the early feeding period (*P* = .004) and further increased 0.35 µm after late feeding (*P* = .078). It suggests that these fat droplets have more than doubled their fat content between T18 and T22. On the other hand, the fat content per adipocyte measured by ORO staining showed an increase in OD value 0.7 after early feeding (*P* < .001), which was limited to a 0.4 OD increase after the late feeding (*P* = .007). Fat droplets can grow by uptake of triglycerides into the cells and by fusion with other droplets.^32^ The lower number of fat droplets, the increase in diameter of the biggest droplets and the lower increase in fat content during the late phase seems in line with an increased contribution of fusion to the enlargement of fat droplets during the late feeding period.

### 3.2 Proteome and secretome changes during the early and late feeding period

In total, 1124 proteins were identified from cell lysates and 1264 proteins from collection medium, of which 393 cellular proteins and 246 secreted proteins were finally selected due to the lower missing value rate as well as secretome validation (Table S1). We then characterized the alterations of the proteome and secretome in the early and late feeding phase. After the early 4‐day feeding (T14‐T18), 82 cellular proteins (Table S2) and 62 secreted proteins were differentially expressed (Table S3). When it comes to the late 4‐day feeding period (T18‐T22), 47 cellular proteins (Table S2) and 63 secreted proteins (Table S3) significantly changed.

### 3.3 Functional analysis of proteins changed during T14‐T18

**FIGURE 2:**
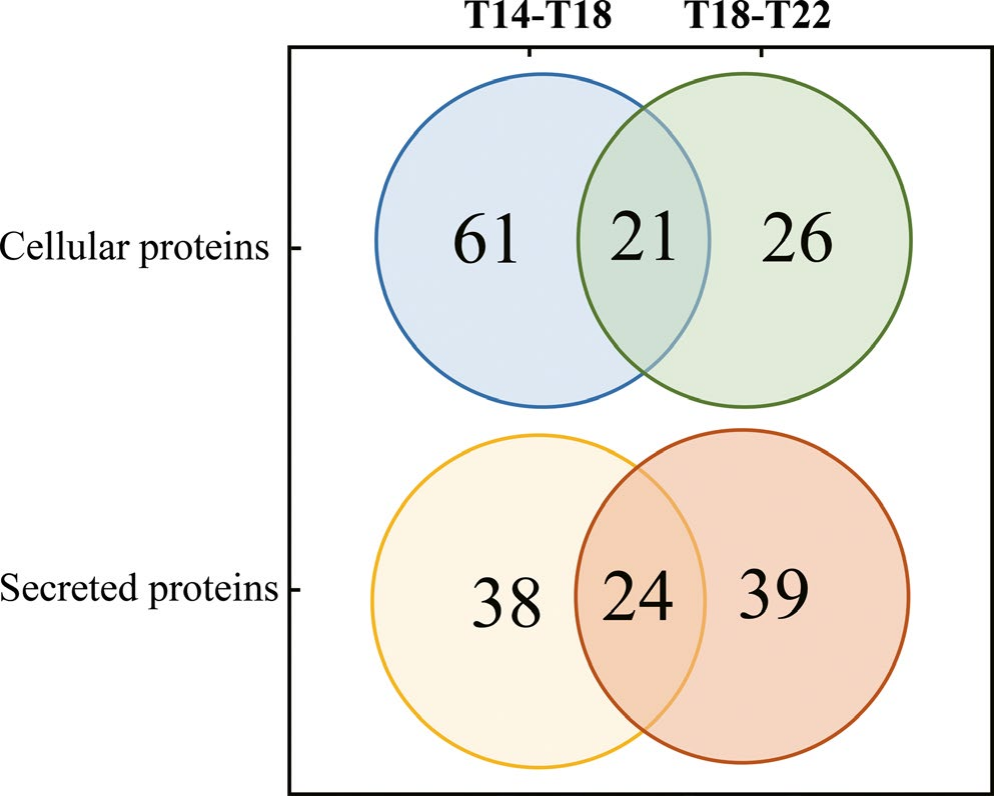
Subgroups of differentially changed proteins during early feeding (T14‐T18) and late feeding (T18‐T22) phase. For the cellular proteins, there were 82 proteins differentially expressed during early feeding and 47 during late feeing, of which 21 cellular proteins were overlapping. For the secretome, there were 62 proteins differentially expressed during early feeding and 63 during late feeding, of which 24 secreted proteins were overlapping

Out of the 82 differential cellular proteins, 61 were unique for the early feeding period (T14‐T18 (Figure 2), of which 20 proteins were up‐regulated and 41 proteins down‐regulated. In parallel, regarding the 62 differentially secreted proteins, 38 were unique for the early feeding period, of which 35 proteins were up‐regulated and only three proteins were down‐regulated.

**FIGURE 3:**
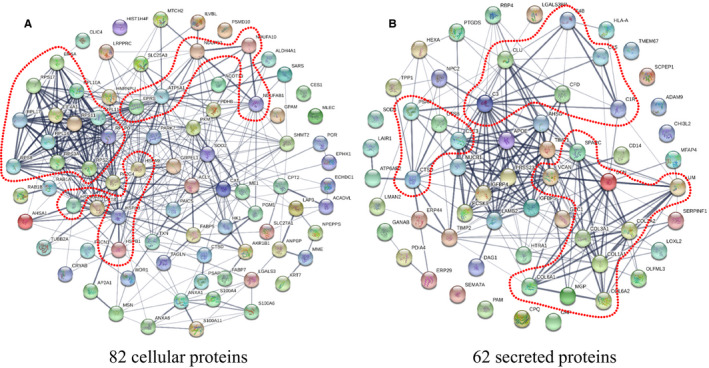
Functional clusters of differential proteins during early feeding (T14‐T18). A, The 82 significantly changed cellular proteins during the early feeding period. B, The 62 significantly changed secreted proteins during the early feeding period. Potential clusters are indicated by a dashed line

Cluster analysis by STRING was performed of the 82 cellular and 62 secreted proteins, respectively (Figure 3). For the cellular proteins, a cluster of ribosomal proteins together with EIF4A1 and EIF5A showed up clearly. This cluster was linked to two smaller clusters, one formed by heat shock proteins (HSP90AB1, HSPA9, HSPA8, HSPB1) with HSPA8 as the node, and the other involving proteins for mitochondrial energy production (NDUFA3, NDUFAB1, NDUFS3, ATP5A1) linked through the ribosome cluster via EPRS (Figure 3A). For the secreted proteins, the predominant cluster was related to extracellular matrix modulation with collagen fibril formation (COL1A1, COL1A2, COL3A1, COL6A1, COL6A2, DCN, LUM, SPARC, VCAN, MGP). In addition, a cluster of complement factors was also observed (C1R, C1S, C3, C4B, CFD, CLU) and a small cluster with secreted lysosomal cysteine peptidases (CTSB, CTSD, CST3, PSAP) (Figure 3B).

Pooling the 82 cellular proteins and 62 secreted proteins, functional analysis using DAVID revealed 20 enriched pathways. The highest scoring pathways were ‘ribosome’ and ‘ECM‐receptor metabolism’ (Table 1).

**TABLE 1:** Pathways of significantly changed cellular and secreted proteins

Sublist	KEGG pathways of the early feeding phase_(T14‐T18)	Count	*P*‐value	Benjamini
1	Ribosome	10	4.9E‐5	6.1E‐3
2	**ECM‐receptor interaction**	7	7.7E‐4	4.6E‐2
3	Legionellosis	5	4.7E‐3	1.8E‐1
4	**Staphylococcus aureus infection**	5	4.7E‐3	1.8E‐1
5	**Protein digestion and absorption**	6	5.0E‐3	1.4E‐1
6	Amoebiasis	6	1.1E‐2	2.4E‐1
7	Complement and coagulation cascades	5	1.1E‐2	2.1E‐1
8	Carbon metabolism	6	1.4E‐2	2.2E‐1
9	Pertussis	5	1.5E‐2	2.0E‐1
10	Biosynthesis of antibiotics	8	1.7E‐2	2.1E‐1
11	**Lysosome**	6	1.8E‐2	2.0E‐1
12	**Protein processing in endoplasmic reticulum**	7	2.0E‐2	2.0E‐1
13	Renin‐angiotensin system	3	3.4E‐2	3.0E‐1
14	Metabolic pathways	23	4.7E‐2	3.7E‐1
15	Glycolysis/ Gluconeogenesis	4	5.2E‐2	3.8E‐1
16	PPAR signalling pathway	4	5.2E‐2	3.8E‐1
17	Galactose metabolism	3	5.5E‐2	3.7E‐1
18	Alzheimer's disease	6	6.1E‐2	3.9E‐1
19	Antigen processing and presentation	4	7.1E‐2	4.2E‐1
20	Systemic lupus erythematosus	5	8.9E‐2	4.7E‐1
21	Pyruvate metabolism	3	9.1E‐2	4.6E‐1
22	Huntington's disease	6	9.5E‐2	4.6E‐1

Sublist	KEGG pathways of late feeding phase_(T18‐T22)	Count	*P*‐value	Benjamini
1	**Protein processing in endoplasmic reticulum**	9	8.6E‐5	1.0E‐2
2	**Lysosome**	7	5.5E‐4	3.3E‐2
3	**ECM‐receptor interaction**	6	8.4E‐4	3.3E‐2
4	Focal adhesion	7	8.1E‐3	2.2E‐1
5	Pathogenic Escherichia coli infection	4	9.3E‐3	2.0E‐1
6	Shigellosis	4	1.7E‐2	2.9E‐1
7	Bacterial invasion of epithelial cells	4	2.9E‐2	4.0E‐1
8	**Protein digestion and absorption**	4	3.9E‐2	4.5E‐1
9	PI3K‐Akt signalling pathway	7	7.4E‐2	6.4E‐1
10	Leucocyte transendothelial migration	4	7.5E‐2	6.1E‐1
11	**Staphylococcus aureus infection**	3	7.8E‐2	5.9E‐1

Note

Pathways were analysed by DAVID. Pathways in bold are overlapping during the early and late feeding period.

### 3.4 Functional analysis of proteins changed during T18‐T22

Regarding the late feeding period (T18‐T22), 26 out of 47 cellular differential proteins were unique for the prolonged feeding period (Figure 2), of which 18 proteins were up‐regulated and 8 proteins were down‐regulated. Of the 63 secreted differential proteins, 39 proteins merely showed up during the late feeding period, of which 23 proteins were up‐regulated and 16 proteins were down‐regulated. Notably, according to the subcellular localization recorded in UniProt,^33^ 10 out of the 47 proteins were cytoskeletal proteins and 8 of those changed significantly during the late period (PFN1, SEPT2, RHOA, SPTBN1, STOM, TUBA1C, MVP and VCL).

**FIGURE 4:**
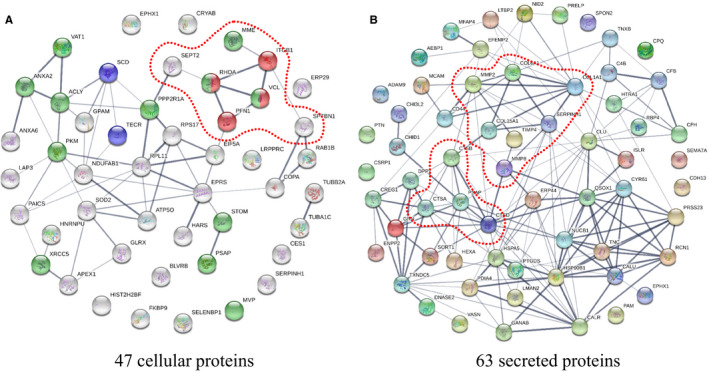
Functional clusters of proteins significantly changed during late feeding (T18‐T22). A, The 47 differentially expressed cellular proteins during the late feeding period. B, The 63 differentially expressed secreted proteins during the late feeding period. Potential clusters are indicated by a dashed line

Using STRING, the 47 cellular and 63 secreted proteins were arranged into functional clusters. As can be seen in Figure 4A, only a small cluster of cytoskeletal proteins linked to focal adhesion was observed with the cellular proteins (MME, RHOA, SEPT2, FPN1, SPTNB1, VCL, ITGB). For the secreted proteins, the most obvious cluster was collagen and collagen modification (COL1A1, COL6A1, COL15A1, MMP2, MMP8, TIMP4, SERPINH1) that linked to the small cluster with lysosomal peptidases (CTSA, CTSB, CTSD, PSAP) (Figure 4B).

After pooling the cellular and secreted proteins, the outcome of analysis with DAVID revealed as most significantly enriched pathways ‘protein processing in endoplasmic reticulum’, ‘lysosome’, ‘ECM‐receptor interaction’ and ‘focal adhesion’ (Table 1).

### 3.5 Comparing metabolic processes between early and late feeding

**FIGURE 5:**
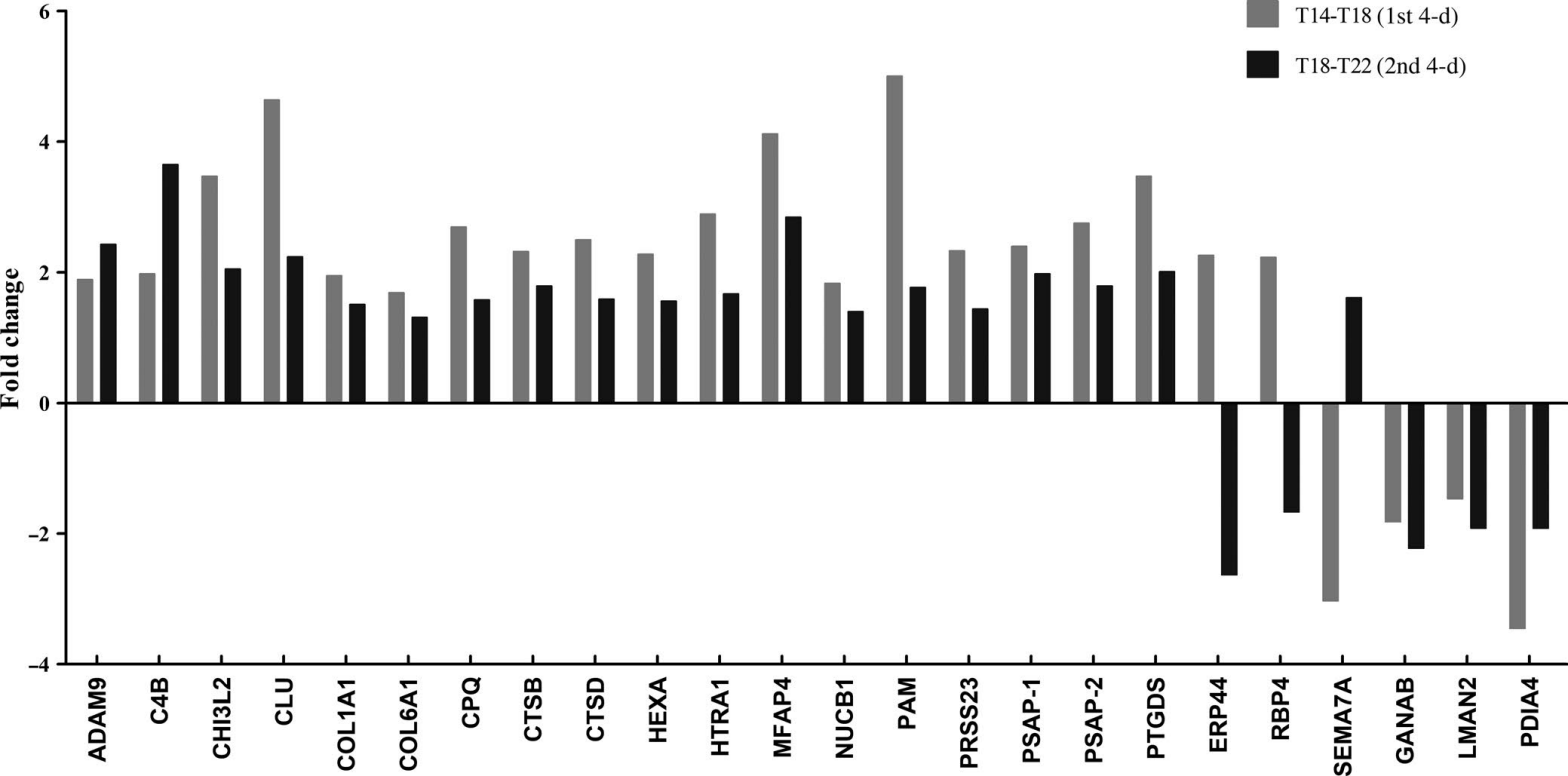
Secreted proteins (n = 24) which significantly changed during both the early (T14‐T18) and late (T18‐T22) feeding periods

Of the differentially secreted proteins of T14‐T18, 92% was up‐regulated (35/38), whereas for T18‐T22 this was 56% (23/39). However, of the 24 overlapping proteins, the vast majority was up‐regulated during both periods. Notably, 20 of the 24 proteins had a significant but lower fold change (FC) during T18‐T22 than during T14‐T18 (Figure 5). The four proteins that behaved differently were ADAM9, C4B, SEMA7A and PDIA4. It suggests that there is a levelling off of protein secretion over time.

Proteins involved in the metabolism of glucose and fatty acids which significantly changed during the early feeding or late feeding period are given in Table 2. Overall, 16/20 proteins (80%) changed during T14‐T18 whereas 7/20 proteins (35%) during T18‐T22. Only three proteins were differentially regulated in both periods. Overall this suggests that more metabolic regulation is happening during the early period, and that over time different regulatory mechanism are active. During T14‐T18, seven proteins were identified related to the glucose metabolism. Another seven proteins play a role in lipid metabolism with the highest change for CPT2, which was 1.41 × down‐regulated suggesting a decreased import of fatty acids into the mitochondria. During T18‐T22, only two of the seven proteins of the glucose metabolism changed with a 2.2 × up‐regulation for ACLY. Four proteins of the lipid metabolism were observed to change including a 1.34 × down‐regulation of GPAM and a 1.21 × down‐regulation of NDUFAB1.

**TABLE 2:** Changes of metabolic proteins during early and late feeding period

Items	T14‐T18	T18‐T22	Protein symbol	Metabolism
*Glucose metabolism*				
Hexokinase‐1	↓	≡	HK1	Glycolysis: conversion of glucose to glucose‐1‐P
Pyruvate kinase PKM	↑	↑	PKM	Glycolysis: conversion of PEP to pyruvate
Pyruvate dehydrogenase E1 component subunit beta, mitochondrial	↑	≡	PDHB	Mitochondrial conversion of pyruvate to acetyl‐coA
Phosphoglucomutase‐1	↓	≡	PGM	Glycogen turnover: reversible conversion of glucose‐1‐P to glucose‐6‐P
ATP‐citrate synthase	↓	↑↑	ACLY	Cytoplasmic production of acetyl‐coA from citrate
NADP‐dependent malic enzyme	↑	≡	ME1	Cytoplasmic conversion of malate to pyruvate and NADPH for FA synthesis
Ethylmalonyl‐CoA decarboxylase	↑	≡	ECHDC1	Conversion of ethylmalonyl‐coA to butyryl‐coA and acetyl‐coA (ACACA by‐product)
*Fatty acid metabolism*				
Very long‐chain enoyl‐CoA reductase	≡	↓	TECR	FA synthesis: elongation of long‐chain FA
Acyl‐CoA desaturase	≡	↑	SCD	FA synthesis: desaturation of FA
Glycerol‐3‐phosphate acyltransferase 1, mitochondrial	≡	↓↓	GPAM	TAG synthesis at sn1 site of phosphoglycerate (prefers saturated FA)
Acyl carrier protein, mitochondrial	↓	↓↓	NDUFAB1	FA synthesis, also subunit of complex I
Acyl‐coenzyme A thioesterase 13	↑	≡	ACOT13	Converts Facyl‐coA into FA and CoA (balances FFA and FA‐coA)
Carnitine O‐palmitoyltransferase 2, mitochondrial	↓↓	≡	CPT2	Import of FA into mitochondrial
Very long‐chain specific acyl‐CoA dehydrogenase, mitochondrial	↓	≡	ACADVL	Mitochondrial beta‐oxidation
Long‐chain fatty acid transport protein 1	↓	≡	SLC27A1	Peroxisomal FA to FA‐coA for beta‐oxidation
Fatty acid‐binding protein, epidermal	↓	≡	FABP5	Fatty acid‐binding and transport
Fatty acid‐binding protein, brain	↑	≡	FABP7	Fatty acid‐binding and transport
*Others*				
ATP synthase subunit O, mitochondrial	≡	↑	ATP5O	Complex V ATP production
ATP synthase subunit alpha, mitochondrial	↓	≡	ATP5A1	Complex V ATP production
Catalase	↓	≡	CAT	Peroxisomal hydrogenperoxide conversion

Note

The changes of metabolic proteins were calculated via fold change (FC). During T14‐T18 and FC was calculated by the abundance of T18 ÷ T14. Similarly, during T18‐T22 and FC was calculated by T22 ÷ T18. ↑ means significantly up‐regulated and ↓ means significantly down‐regulated. ≡ means the change was not significant. The double arrow means the FC was above 2 (↑↑) or below 2 (↓↓).

## 4 DISCUSSION

In the present study, we investigated the changes over time of the cellular proteome and of the secretome of human SGBS adipocytes under conditions of high glucose and high insulin. We identified 393 cellular proteins and 246 secreted proteins for further analysis. Pathway analysis, functional clustering analysis, metabolic proteome changes and morphologic characterization of the adipocytes allowed us to determine time‐dependent changes in the molecular and metabolic processes of the adipocytes.

Early feeding of mature adipocytes with high glucose in the medium seemed to promote the production of pyruvate (up‐regulation of PKM, ME1) and of mitochondrial acetyl‐CoA (up‐regulation of PDHB). Cytoplasmic production of acetyl‐CoA was decreased (down‐regulation of ACLY). Regarding the lipid metabolism, seven proteins were differentially expressed during the early feeding period. Changes of protein abundances were in line with a lowering of beta‐oxidation in mitochondria (down‐regulation of CPT2, ACADVL and NDUFAB1) and in peroxisomes (down‐regulation of SCL27A1 and CAT) accompanied by increased de‐esterification of fatty acyl‐CoA (up‐regulation of ACOT13). Altogether this indicates that during T14‐T18 the adipocytes prefer to use glucose as fuel and prefer to store the lipids in agreement with the increase of the lipid content and size of the biggest fat droplets. The 10 clustered ECM proteins in the secretome with the addition of LAMB2 and DAG1 are all up‐regulated, which is in line with an increased fat load causing vulnerability of adipocytes for mechanical disruption and the need to synthesize a protective ECM. Despite the increased capacity of pyruvate production, down‐regulation of HK1 (FC = −1.07, *P* = .008) suggests a down‐grading of glycolysis, which seems to be accompanied by a decreased mitochondrial production of ATP (down‐regulation of NDUFA3, NDUFAB1, NDUFS3, ATP5A1). A reduction of the available energy could explain the 7%‐28% down‐regulation of translation indicated by the clustered ribosomal proteins, translation initiation factors EIF4A1 (FC = −1.13, *P* < .001) and EIF5A (FC = −1.07, *P* = .02), and the down‐regulation of SARS (FC = −1.09, *P* = .03). Under those circumstances, the increased abundance of ECM proteins could either be the consequence of increased post‐translational processing of precursor proteins induced by high insulin^34^ or may represent a funnelling of translational energy consumption towards the production of ECM proteins essential for survival of the adipocytes.^35^, ^36^ Because several important collagen‐modifying enzymes (PCOLCE, PCOLCE2, P3H1, P4HA4, PLOD1, PLOD3) do not significantly change during the early nor the late feeding, a funnelling of the translational energy consumption seems more likely, but this has to be further investigated.

The adjusted levels of most of the glucose converting enzymes do not further change during T18‐T22. Only the final step of glycolysis by PKM is further increased. Notably, ACLY is significantly up‐regulated (FC = 2.20, *P* = .05), which could boost the production of cytoplasmic acetyl‐CoA. This can be used by the adipocytes for de novo fatty acid synthesis by the enzyme acetyl‐CoA carboxylase (ACACA, ACACB). Both ACACA and ACACB do not change significantly during each of the feeding periods. Yet, some degree of de novo fatty acid synthesis is supported by the up‐regulation of ECHDC1 during T14‐T18, which breaks down ethylmalonyl‐coA as a by‐product of ACACA, and by the up‐regulation of SCD during T18‐T22. On the other hand, the abundance of fatty acid synthase (FASN) did not change during the early and late feeding period (FC = −1.01, *P* = .75 and FC = 1.03, *P* = .70, respectively). Notably, during the late feeding period the more advanced steps of lipid synthesis seem to be reduced by down‐regulation of TECR (FC = −1.18, *P* = .03) which catalyses the final step of very long‐chain fatty acid synthesis, and of GPAM (FC = −1.34, *P* = .01) which catalyses the first step of triglyceride synthesis, for which it prefers saturated fatty acids. The reduced production of triglycerides is in line with the lower increase of the lipid content of the adipocytes. Because the fat droplets still grow considerably bigger during this period, there seems to be a transition from uptake of lipids into the droplets to the fusion of small to big droplets.^37^ In line with this, far less ECM proteins were up‐regulated during this period (COL1A1, COL6A1, COL15A1, NID2) in a cluster with up‐regulated ECM‐processing enzymes (MMP2, MMP8, TIMP4, SERPINH1). In addition, over time the small cluster with lysosomal proteases changed towards a more active profile. During early feeding, CTSB and CTSD cluster with CST3, which is an inhibitor of cysteine proteases, and with PSAP which can ameliorate the inhibitory activity of CST3.^38^ In the late stage, CTSB, CTSD and PSAP are further up‐regulated but the inhibitor CST3 is not. Therefore, it is no longer part of the cluster, but another protease CTSA becomes part of it. It suggests that during the late feeding phase the ECM does not anymore have to grow, but needs to be maintained.

Since adipocyte overgrowth in vivo may be accompanied by the development of insulin resistance,^12^, ^39^, ^40^ we checked the insulin sensitivity of our cells by determining the phospho‐AKT/AKT ratio at T14, T18 and T22. The ratio at T22 was reduced by 35% as compared to T14. Although this was not significant (*P* = .14; Figure S1), it suggests that the cells are developing insulin resistance over time. Notably, the above‐mentioned measurement does not provide the real cell ability to respond to insulin as we did not compare it to insulin‐starved cells. Therefore, it remains possible that already in the first four days there was a reduction of insulin sensitivity. This could explain why two insulin‐stimulated enzymes, ACLY and HK1, are down‐regulated during T14‐T18. Partial insulin resistance could reduce the production of cytoplasmic acetyl‐CoA from citrate released by the mitochondria, where it is produced from pyruvate. As such, lipid production is already reduced in the early feeding stage, which could be regarded as a cellular response to limit overgrowth.

Notably, during both periods of high glucose and high insulin feeding various factors of the complement system were significantly altered in abundance. During T14‐T18, we found C1R, C1S, C3, CFD, C4B and CLU strongly up‐regulated (FC = 1.9‐4.6), whereas during T18‐T22 up‐regulation of C3, C4B and CLU (FC = 1.6‐3.7) continued together with up‐regulation of CFB (FC = 3.2) and down‐regulation of CFH (FC = −1.7). For the moment, we do not know the biological relevance of these changes, but it is tempting to suggest that it somehow relates to the increased inflammatory nature of hypertrophic AT.^41^, ^42^

It should be noticed that the culture conditions that we used here with high glucose and high insulin are not directly comparable with in vivo conditions observed in cases of glucose intolerance or diabetes. However, our in vitro system could shed light on the changes of adipocytes as they move from the mature state (T14) to a state with maximum lipid load (T22). As such, the observed changes could mimic what happens in vivo during the development of adipocyte hypertrophy.

In summary, in the early stage the adipocytes seem to prefer pyruvate as energy source, whereas beta‐oxidation is down‐regulated supporting lipid loading. Also, glycolysis is being limited which is accompanied by reduction of protein translation. Over time, lipid loading of the cells reduces paralleled by a reduction of the triglyceride synthesis capacity. Consequently, fusion becomes relatively more important for growth of lipid droplets during the late stage. Nevertheless, ECM formation is promoted probably to protect the lipid‐loaded cells against mechanical rupture.

## 5 CONCLUSIONS

In conclusion, the present in vitro system provides insight into the molecular and metabolic changes of mature adipocytes under conditions of high glucose and insulin, which may help to understand the process of in vivo adipocyte hypertrophy during the development of obesity. Here, we have used SGBS cells, but similar studies can now be performed in primary adipocytes or induced adipose tissue‐derived stem cells (iASCs) of both subcutaneous and visceral adipose tissue to link depot‐specific proteome and secretome changes of overgrowing adipocytes to metabolic consequences in humans.

## CONFLICT OF INTEREST

The authors declare no conflict of interest.

## AUTHOR CONTRIBUTIONS

**Qi Qiao:** Writing‐original draft (equal). **Freek G. Bouwman:** Software (equal); Writing‐review & editing (equal). **Johan Renes:** Methodology (equal); Writing‐review & editing (equal). **Edwin C. M. Mariman:** Conceptualization (equal); Supervision (equal); Writing‐review & editing (equal).

## Supporting information

Figure S1

Tables S1‐S3

## Data Availability

Data can be obtained from the corresponding author on request.
