# Supplementary material for: An in vitro model for hypertrophic adipocytes: Time‐dependent adipocyte proteome and secretome changes under high glucose and high insulin conditions
Source: J Cell Mol Med. 2020 Jul 3;24(15):8662–73. doi: 10.1111/jcmm.15497 (PMC7412416; doi:10.1111/jcmm.15497)
Supplement: Supplementary file 1 — Figure S1 [file JCMM-24-8662-s001.docx]

**List of supplemental files**

**1. Supplemental Figure and legend:**


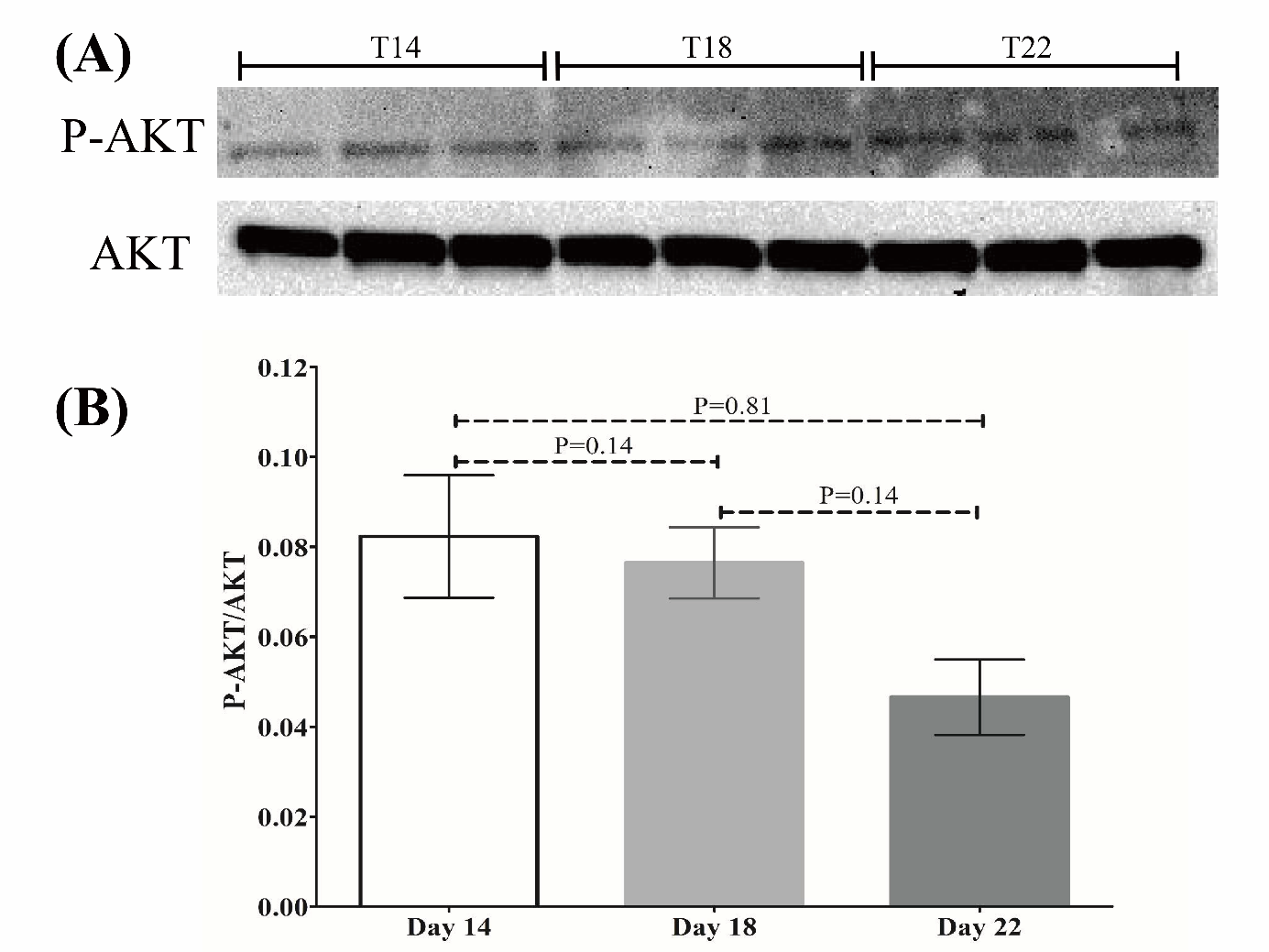
 **Supplemental Figure S1.** The expression of AKT and phospho-Akt (P-AKT) at T14, T18 and T22. **(A):** The ratio of P-AKT/AKT (mean ± SEM) at T14, T18 and T22. **(B):** Statistical analysis was performed by using the dependent T-test, the ratios were not significantly different with a cut-off for significance of p < 0.05. SEM: the standard error of the mean.
